# Supplementary material for: The Impact of Theta-Burst Stimulation on Cortical GABA and Glutamate in Treatment-Resistant Depression: A Surface-Based MRSI Analysis Approach
Source: Front Mol Neurosci. 2022 Jul 13;15:913274. doi: 10.3389/fnmol.2022.913274 (PMC9328022; doi:10.3389/fnmol.2022.913274)
Supplement: Supplementary file 1 [file Data_Sheet_1.pdf]

Supplementary Material

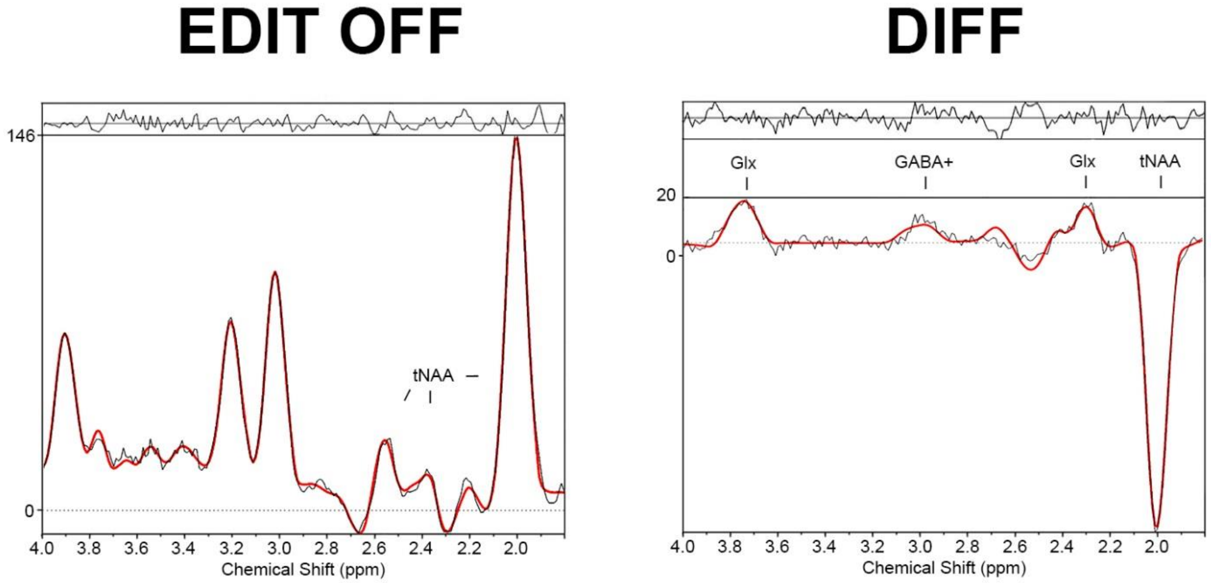

Supplement figure 1: Exemplary unedited (EDIT OFF) and edited (DIFF) spectra derived from the caudal middle frontal area showing peaks of total N-acetylaspartate (tNAA), a combination of GABA and macromolecules (=GABA+) and a combined signal of glutamate and glutamine (=Glx).

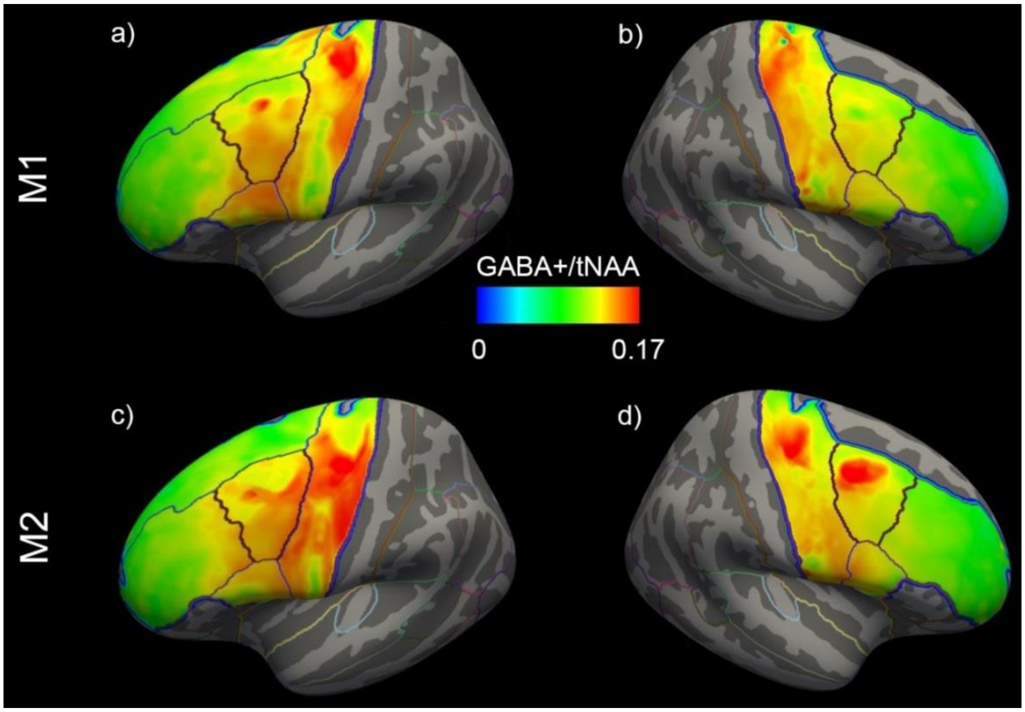

Supplement figure 2: Mean distribution of GABA+/tNAA ratios before (=M1) and after (=M2) the treatment period of the left (a and c) and right (b and d) hemisphere. tNAA = total N-acetylaspartate; GABA+ = a combination of GABA and macromolecules.

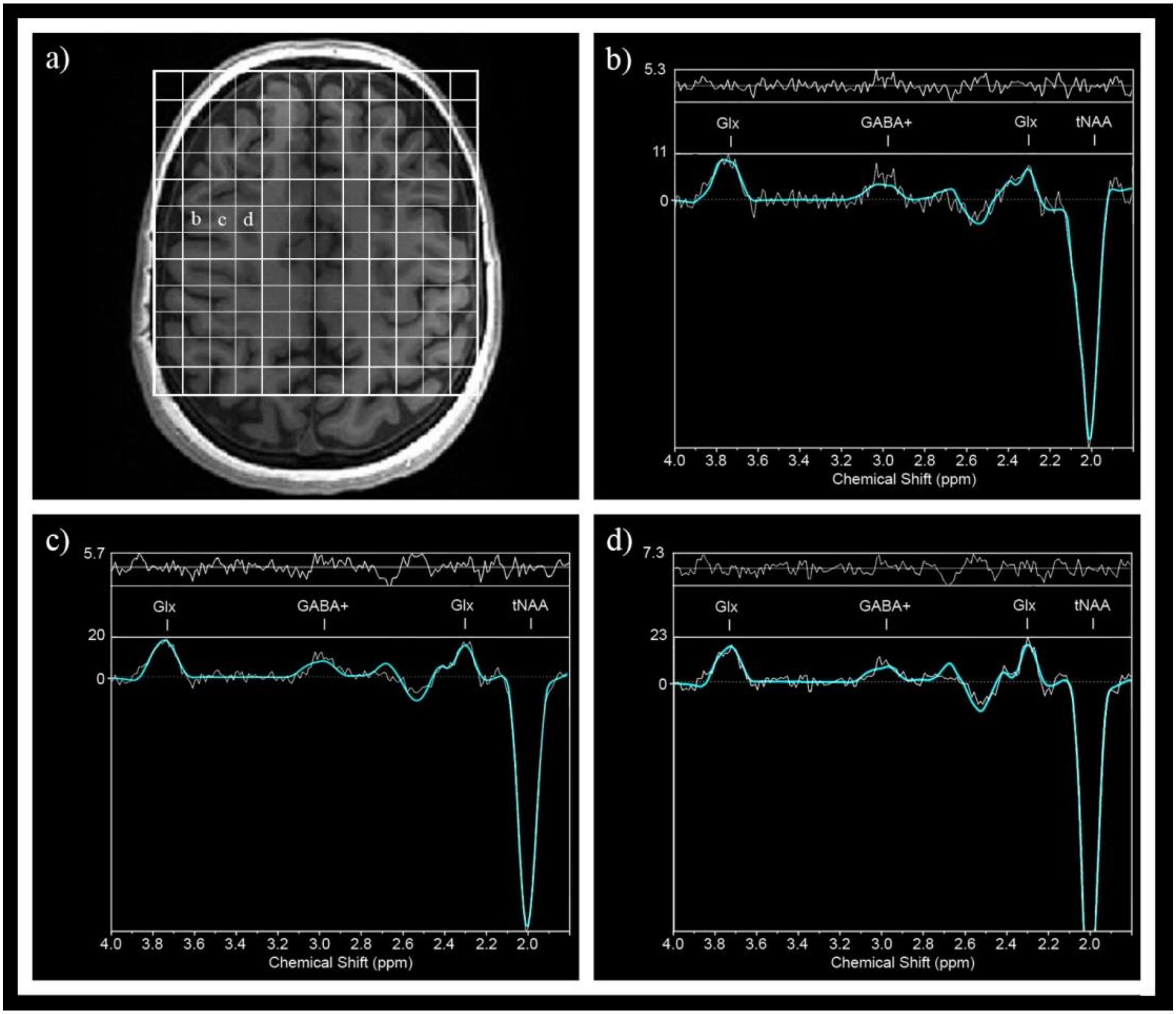

Supplement figure 3: Example series of spectra in cortical regions demonstrating the effects of lipid suppression on spectral quality. Stable quantification of skull-near (b) to inner cerebral (c,d) spectra is shown. Voxel positions of spectra (b,c,d) are marked in the voxel grid (a). tNAA = total N-acetylaspartate; GABA+ = a combination of GABA and macromolecules, Glx = glutamate + glutamine.

Supplement table 1: Detailed medication of the TRD patients (with dosages in milligram where documented)

subject	medication
1	sertraline 100mg, pregabalin
2	venlafaxine 150mg, bupropion 150mg, lamotrigine 100mg, pregabalin 225mg, olanzapine 2.5mg, amitriptyline 20mg
3	none
4	sertraline 200mg, escitalopram 5mg
5	none
6	sertraline 100mg
7	escitalopram 10mg, mianserin 15mg
8	escitalopram 30mg
9	venlafaxine 150mg, lithium 450mg
10	milnacipran
11	melitracen
12	none
